# Diurnal Circadian Lighting Accumulation Model: A Predictor of the Human Circadian Phase Shift Phenotype

**DOI:** 10.1007/s43657-021-00039-6

**Published:** 2022-02-11

**Authors:** Dandan Hou, Caixin Lin, Yandan Lin

**Affiliations:** 1 Institute for Electric Light Sources, School of Information Science and Technology, Fudan University, Shanghai, 200438 China; 2 Institute of Future Lighting, Academy for Engineering and Technology, Fudan University, Shanghai, 200433 China; 3 Human Phenome Institute, Fudan University, Shanghai, 201203 China

**Keywords:** Circadian phase phenotype, Diurnal light exposure, Circadian phase shift model

## Abstract

Light is an important external factor that affects human circadian rhythms. This study aimed to explore the effects of different dimensions of diurnal light exposure on the physiological circadian phase shift (CPS) of the human body. A strict light exposure experiment with different timing schemes (8:00–12:00, 13:00–17:00, 18:00–22:00), durations (4 h, 8 h) and effective circadian stimulus levels (circadian stimulus: 0.35, 0.55) was performed in an enclosed laboratory. Fourteen participants, including seven males and seven females, with a mean age of 24.29 ± 2.43 (mean ± standard deviation), participated in this experiment and experienced all six lighting schemes. The results showed that both time factor (*F*_3,40_ = 29.079, *p* < 0.001, the power of the sample size = 0.98) and circadian stimulus levels (*T*_20_ =  − 2.415, *p* = 0.025, the power of sample size = 0.76) significantly affect the CPS. On this basis, a diurnal circadian lighting accumulation (DCLA)—CPS model was proposed in the form of the Boltzmann function, and was validated by experimental data with high correlation (*R*^2^ = 0.9320, RSS = 0.1184), which provides strong support for rationally arranging the light level at different times of the day.

## Introduction

Light is an important environmental factor that affects the physiological phenotype of the human body (Abbas et al. 2006; Vetter et al. 2021), such as brain activity (Yuan et al. 2021), body temperature (Lok et al. 2019), neuroendocrine function (Leproult et al. 1997), emotion (Yan et al. 2019) and circadian rhythm (Duffy and Wright 2005; Duffy and Czeisler 2009; Tähkämö et al. 2019). Proper lighting exposure during the daytime could stabilize the circadian rhythm phase, which then has a positive impact on mood and sleep (Figueiro et al. 2017). However, inappropriate use of light at night (LAN) could cause severe human health concerns (Pauley 2004). For example, artificial light exposure with short wavelengths (blue) at night was evidenced to have a positive relationship with breast cancer, circadian rhythm disruption and abnormal melatonin suppression (Rybnikova and Portnov 2018). Hence, the influence of diurnal light exposure on the circadian phase needs to be studied to help people choose the right level of light at the right time to remain healthy.

The time factor of light exposure, including timing and duration, is an important factor that affects the circadian phase shift (CPS). For the timing effects, Minors et al. (1991) proposed a circadian phase response curve (PRC) to describe the effect of 3-h bright light exposure at different times on the amplitude and direction of CPS. Although the PRC laid the foundation for subsequent research, it is difficult to apply because it is based on a vague report of light level and the curve is very complicated. In addition, Carrier and Dumont (1995) investigated the effect of bright light exposure at different times of the day on body temperature rhythm and demonstrated that light exposure in the morning (CPS = −1.23 h) and afternoon (CPS = −0.48 h) could advance the circadian phase, while light exposure at night (CPS =  + 1.62 h) showed a delay in the circadian phase. This was consistent with the conclusion of Minors et al. (1991) and Revell et al. (2006), which support that light exposure with different timing schemes has an influence on the amplitude and direction of the CPS. For duration effects, Chang et al. revealed that the light duration has a significant impact on the rhythmic phase shift and subjective sleepiness. As the light duration increased, the circadian phase response exhibited nonlinear characteristics (Chang et al. 2012), which indicated that with the accumulation of light stimulation, the CPS amplitude would not increase endlessly, but a response compression phenomenon occurred, and the CPS had an upper limitation even though the light accumulation was large enough. All these conclusions have led us to comprehensively consider the effects of timing and duration as a light exposure response curve in the time dimension. Moreover, the current research studies have not provided enough data that can be used to analyse the combined effects of these two factors at the same time.

The light stimulus level is another essential factor that affects the CPS. Previous studies have proven that higher illuminance levels (Dewan et al. 2011) or more blue light components in the spectrum (normally higher correlated colour temperature (CCT)) (Smith and Eastman 2009; Higuchi et al. 2016) could trigger greater CPSs. However, these parameters used to describe the light stimulus level are calculated based on the response of the visual pathway in the human eye instead of the response of the non-visual pathway that acts on the human circadian rhythm. Souman et al. (2018) verified that spectral tuning with different melanopic illuminance (54.6 lx and 188.8 lx) and melanopic efficacy (0.30 and 1.07) could also cause strongly different reduction in melatonin suppression at night (*p* < 0.05), although without changing illuminance level (175 lx) or colour temperature (2700 K). Therefore, the parameters describing the influence of light on circadian rhythm should be optimized to which indicators are calculated based on the response sensitivity of a non-visual pathway such as intrinsically photosensitive retinal ganglion cells (ipRGCs) (Berson et al. 2002). Circadian light (CL_A_) and circadian stimulus (CS) (Rea and Figueiro 2018), developed by Rea and Figueiro, and melanopic illuminance (*E*_z_) (Lucas et al. 2014), recommended by the International Commission on Illumination (CIE) (CIE 2017, 2018), are all better choices for describing the circadian stimulus level. The equations of *E*_z_, CL_A_, and the circadian stimulus (CS) (Rea et al. 2005) are shown in Eqs. (1)–(3).

1 $$E_{z} = K_{{\text{N}}} \int\limits_{360}^{830} {E_{e,\lambda } (\lambda )} N_{z} (\lambda ){\text{d}}\lambda ,$$

where $K_{{\text{N}}}$ is the non-visual spectral efficacy constant, which equals 73,000 z-lm/W; $E_{e,\lambda } (\lambda )$ represents the spectral irradiance; and $N_{z} (\lambda )$ is the melanopsin (z-opic) spectral efficiency function, which can be found in CIE TN003-2015 (CIE 2015).

2 $${\text{CL}}_{{\text{A}}} = \left\{ \begin{gathered} 1548\left[ {\int {Mc_{\lambda } E_{\lambda } {\text{d}}\lambda + \left( {a_{b - y} \left( {\int {\frac{{S_{\lambda } }}{{mp_{\lambda } }}E_{\lambda } {\text{d}}\lambda - k\int {\frac{{V_{\lambda } }}{{mp_{\lambda } }}E_{\lambda } {\text{d}}\lambda } } } \right) - a_{{{\text{rod}}}} \left( {1 - e\frac{{ - \int {V^{\prime}_{\lambda } E_{\lambda } {\text{d}}\lambda } }}{{{\text{RodSat}}}}} \right)} \right)} } \right] \hfill \\ {\text{ if }}\int {\frac{{S_{\lambda } }}{{mp_{\lambda } }}E_{\lambda } {\text{d}}\lambda - k\int {\frac{{V_{\lambda } }}{{mp_{\lambda } }}E_{\lambda } {\text{d}}\lambda } }  0 \hfill \\ 1548\int {Mc_{\lambda } E_{\lambda } {\text{d}}\lambda \;{\text{if}}\;\int {\frac{{S_{\lambda } }}{{mp_{\lambda } }}E_{\lambda } {\text{d}}\lambda - k\int {\frac{{V_{\lambda } }}{{mp_{\lambda } }}E_{\lambda } {\text{d}}\lambda } } \le 0 \, } \hfill \\ \end{gathered} \right.,$$

where 1548 is calculated by the standard light source CIE A (2856 K) of 1000 lx (${\text{CL}}_{{\text{A}}} = 1000$); $E_{\lambda }$ represents the spectral irradiance distribution of light sources; and $Mc_{\lambda }$, $S_{\lambda }$, $V_{\lambda }$, and $V^{\prime}_{\lambda }$ are the efficiency functions of melanopsin, S-cone, photopically luminous and scotopically luminous, respectively. $mp_{\lambda }$ represents the macular pigment transmittance, and RodSat is equal to 6.5 W/m^2^, which is the half-saturation constant for rods. In addition, $k$, $a_{b - y}$, and $a_{{{\text{rod}}}}$ are all constants in this function, which equals 0.2616, 0.7000, and 3.3000, respectively (Rea and Figueiro 2018).

3 $${\text{CS}} = 0.7 - \frac{0.7}{{1 + \left( {\frac{{{\text{CL}}_{{\text{A}}} }}{355.7}} \right)^{1.1026} }},$$

where CS is a derivative index of ${\text{CL}}_{{\text{A}}}$, and their functional relationship is shown in Eq. (3). CS is constructed based on nocturnal melatonin suppression from lighting.

A recent study also proved that CL_A_ and *E*_z_ have a high fitting degree in describing the effect of light intervention on the dim light melatonin onset (DLMO) shift (Hou et al. 2021a). However, the illuminance also varied, while the circadian stimulus level (CL_A_ and *E*_z_) changed in their study. Therefore, further research should be conducted to explore the effect of circadian stimulus level on CPS with constant colour temperature and illuminance. Besides, another study (Nagare et al. 2019) investigated the combined effects of illuminance, spectrum and light exposure duration on melatonin suppression at night. The results showed that the lighting condition with lower CS levels needs a longer duration to trigger significant melatonin suppression than the higher CS levels, which implied the combined effects of time factor and circadian stimulus factor were important to affect the CPS.

In this study, we hypothesized that the cumulative amount of effective diurnal lighting, considering timing and duration and effective circadian stimulus level, is the root cause of the rhythmic phase shift. A light exposure experiment with different timing schemes, durations and circadian stimulus levels was performed to explore the effects of time factors and effective light stimulus level factors on the physiological CPS of the human body. Then, according to the multidimensional experimental results, a diurnal circadian light accumulation (DCLA) model was proposed to predict the human CPS.

## Materials and Methods

### Participants

Participants in this study were recruited at Fudan University, which included 14 undergraduate or graduate students, including seven males and seven females, with a mean age of 24.29 ± 2.43 (mean ± SD). Considering that this study was mainly for people with a normal circadian phase and healthy eye and physical conditions, the inclusion criteria were as follows: (1) the outcomes of the morningness-eveningness questionnaire (MEQ) (Horne and Ostberg 1976) belonged to the "intermediate type"; (2) the vision or corrected vision was greater than 1.0; and (3) the circadian rhythm was stable, and the participants were based in Shanghai for at least three months. The exclusion criteria were as follows: (1) colour blindness or weakness; (2) indulgence in drinking alcohol, coffee or tea; (3) serious illness; and (4) sleep disorders or depression. In addition, before the experiment, the participants were informed of the detailed procedures and assessments by written informed consent. The experimental procedure was approved by the School of Life Science, Fudan University (No. BE2042) under the Declaration of Helsinki.

### Experimental Setup

**Fig. 1:**
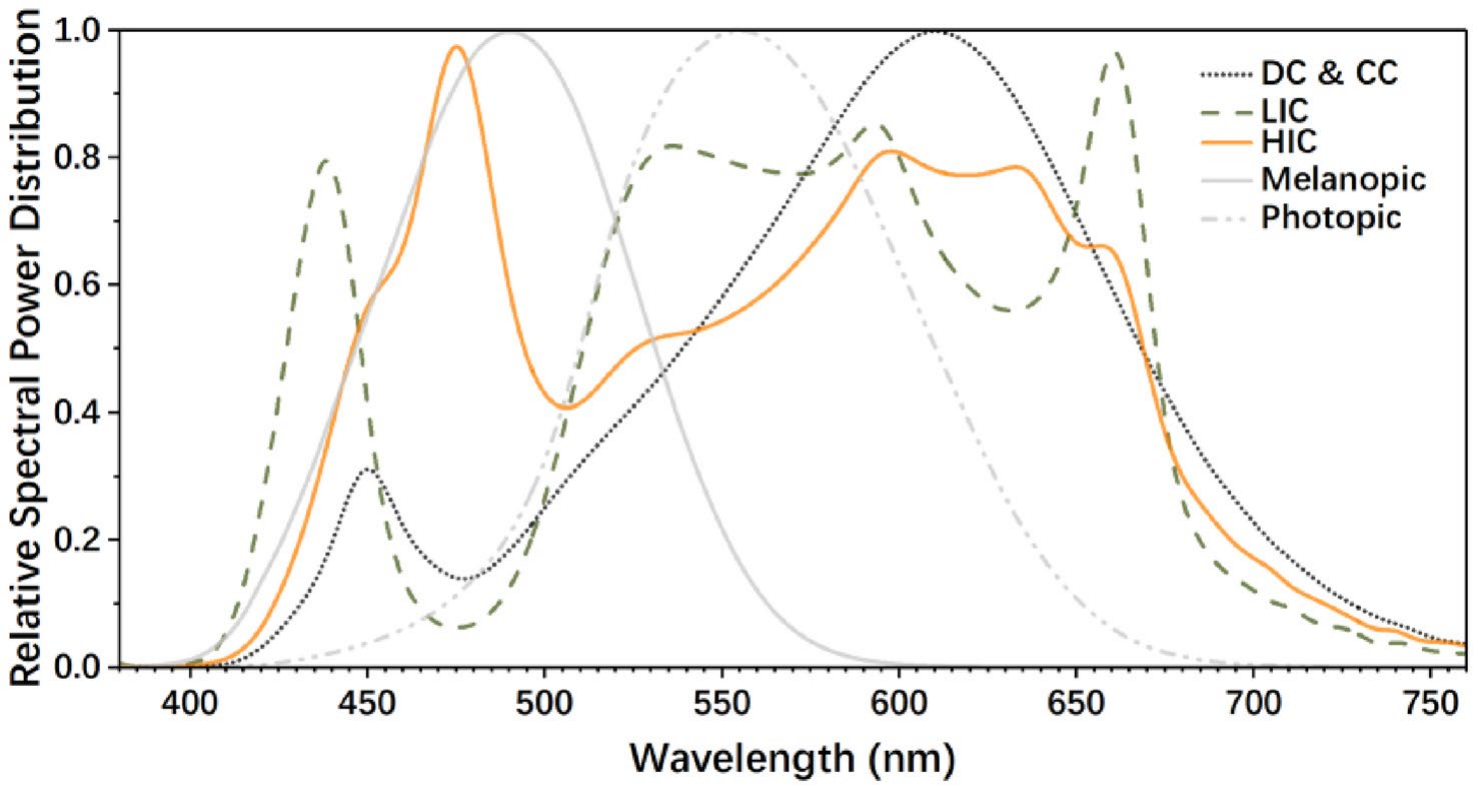
The relative spectral power distribution of the lighting conditions in this study. The photopic and melanopic efficiency functions are based on CIE TN003-2015 (CIE 2015)

Six lighting schemes were designed to investigate the effects of diurnal light exposure timing, duration, and effective circadian stimulus level on the CPS by the orthogonal experimental design method, which was widely used in the multi-dimension experiment to improve the experimental efficiency (Zuo et al. 2016). The schemes were composed of four lighting conditions: (1) dim condition (DC); (2) control condition (CC); (3) low-intervention condition (LIC), and (4) high-intervention condition (HIC). The specific parameters of these conditions are shown in Table 1. Among them, DC was the control condition at night (18:00–22:00), with a low illuminance level (< 10 lx) (Pandi-Perumal et al. 2007) and CS level (< 0.1) (Alzahrani et al. 2021), which ensured that the circadian phase phenotypic and hormone levels would not be disturbed (Zeitzer et al. 2000b; Kozaki et al. 2011). CC was set as the control condition during the daytime (8:00–18:00), and its relative spectral power distribution (SPD) was the same as DC (CS < 0.1), but with an illuminance level of 90 lx (St Hilaire et al. 2012) and maintaining its CS value less than 0.1, which provided basic visual demand but had no effect on circadian rhythm (Alzahrani et al. 2021). For LIC and HIC, spectral tuning technology allowed the two conditions to produce the greatest CS differences under the same colour temperature and illuminance to explore the effective spectral components that affect the circadian rhythm response and generate the largest differences in response. The light levels of LIC and HIC were of a sufficient illuminance level (~ 1000 lx) to trigger the circadian phase resetting response, which has been verified in previous studies (Zeitzer et al. 2000b; Chang et al. 2012). The SPDs of the four conditions are shown in Fig. 1.

**Table 1:** Lighting conditions in this study

Condition	Vertical illuminance (Ev, lx)	Correlated colour temperature (CCT, K)	Chromaticity coordinate (CIE 1931)	Duv	Colour rendering index (CRI, Ra)	Circadian stimulus (CS)	Circadian light (CL_A_)	Melanopic illuminance (*E*_z_, lx)
DC	9.83	2700	(0.4643, 0.4142)	0.0010	86	0.01	7.71	4.14
CC	90.00	2700	(0.4643, 0.4142)	0.0010	86	0.09	69.37	37.91
LIC	987.10	4005	(0.3878, 0.4048)	0.0105	75	0.35	353.97	520.09
HIC	1007.23	4038	(0.3701, 0.3428)	−0.0137	85	0.55	1177.83	937.84

The CS and CL_A_ values in this table were calculated according to the latest research of Rea and Figueiro (2018). The *E*_z_ was calculated by the melanopic response curve in CIE TN003-2015 (CIE 2015)

**Fig. 2:**
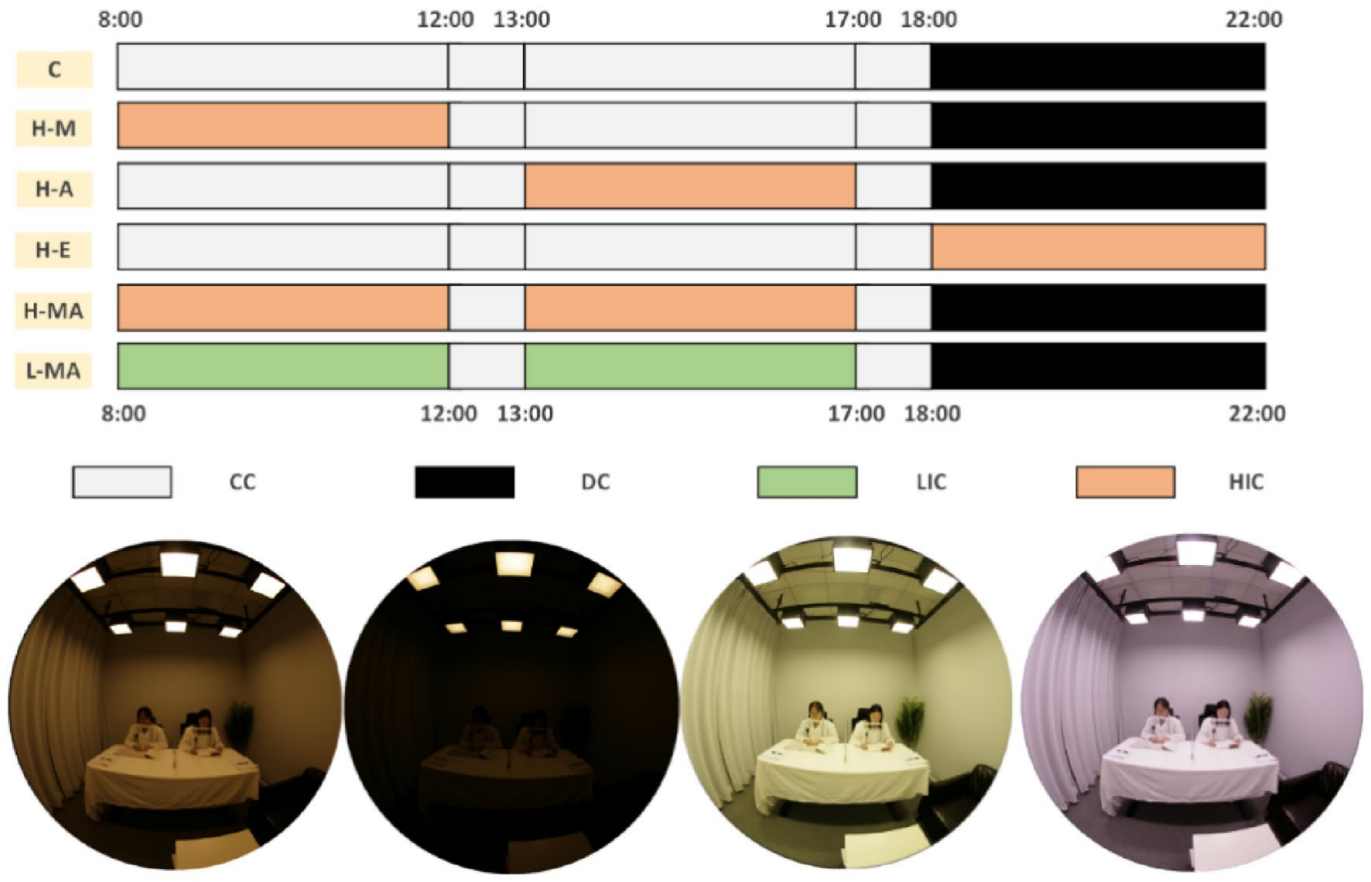
Six lighting schemes and four lighting conditions in this study. CC represents the control condition of lighting, DC is the dim condition, LIC means the low-intervention condition, and HIC represents the high-intervention condition. The words in the six yellow blocks are the names of the following schemes

Six lighting schemes are shown in Fig. 2. The control scheme (C) was set as the control condition to collect the baseline of participants. The lighting conditions are arranged in the CC during the daytime (8:00–18:00) and DC during the evening (18:00–22:00). For the following experimental schemes, the light conditions remained the same as C in all periods without light intervention. Then, the effective CPS outcomes at different time points with a fixed light exposure duration of 5 h in Carrier and Dumont’s research (Carrier and Dumont 1995) and the normal light exposure period (8:00–22:00) for people who were classified as "intermediate type" are referenced. A similar fixed light exposure duration of 4 h for three schemes at different times is as follows: (1) High intervention in the morning (H-M); (2) High intervention in the afternoon (H-A); and (3) High intervention in the evening (H-E). From this design, it is also possible to investigate the effects of timing on the CPS phenotype. In addition, the duration effects could be discussed by the above three timing schemes (4 h) and the special scheme, that is, the HIC lighting condition in both the morning and afternoon (H-MA) schemes (8 h). Another scheme (low intervention in both the morning and afternoon, L-MA) could be compared with H-MA to explore the effects of different CS levels by different spectra with constant colour temperature and illuminance.

The experiment was conducted in a real lit office. The dimensions of the office were L 3.2 m × W 3.0 m × H 2.8 m. Six 11-channel light-emitting diode (LED) cubes (Thouslite tunable LED system, Changzhou Qianming) were positioned to be 2.6 m above the floor to provide the lighting conditions for the experiments. All the objects in this room were neutral colours, except for a plant in the corner. The SPD and illuminance of each condition were measured by a spectrophotometer (CL-500A, Konica Minolta) every 10 min at the eye position of two participants, which was 1.2 m above the floor. A frosted partition was placed on the table between two subjects to avoid the interactive effects between them.

### Procedures

To control the circadian rhythm characteristics, participants were asked to report their daily sleep and waking time recorded by a wristband (Huawei FIT 2, Huawei Device (Dongguan) Co., Ltd., China) (Zhang et al. 2018) from a week before the experiment starting to the last experimental day for a total of 42 days. The data collected from wristbands were not used as the outcomes of the CPS, only for monitoring and confirming whether the participants’ sleep periods were stable during 23:00–7:00 for one week before the day to receive light intervention, controlling regular sleep habits one week before the formal experiment. Each participant experienced all six lighting schemes in a randomized order, at least one week between every two schemes to elute the influence of the previous scheme on the circadian phase.

On each experimental day, each participant was asked to arrive at the laboratory at 7:50 and wore the white lab coat before 8:00, and then take a body temperature monitoring capsule at 8:00. The lighting scheme was conducted during 8:00–22:00, as shown in Fig. 2. During the lighting scheme period, participants needed to stay in the laboratory to avoid other light exposure. They could use self-luminous electronic devices at the lowest luminance for no more than 5 min per hour. To strictly control the light intake on the experimental day, the subjects were required to wear sunglasses before arriving in the laboratory and after leaving the laboratory to reduce uncontrollable light interference.

### Physiological Data Acquisition

The rhythm of core body temperature (CBT) is an important physiological phenomenon of the human circadian rhythm (Kräuchi 2002). Moreover, core body temperature minimum (CBT min) is well documented as a circadian marker (circadian time 0) (Jehan et al. 2017). Compared with the DLMO measurement, CBT can be recorded at a higher sample rate, and thus, the CBT min could be captured more accurately (Lovato et al. 2016). In this study, a continuous core temperature monitoring system (e-Celsius Performance®, BodyCAP) was employed to track the diurnal CBT rhythm of the participants, which was tested by 2011/65/EU and 2014/53/EU (Ref. P060REG001.1), taken by the participants at 8:00 on the experiment day and recorded at 12:00 of the next day to complete data synchronization and reading. The sample interval of the capsule was 30 s. In the process of data collection performed by the capsule, participants were asked to mark special events that might affect body temperature, such as drinking water, eating, and bathing. These markers could provide the basis for the exclusion of outliers in data processing. The accuracy of this system was ± 0.2 °C in the range of 25–45 °C, with a variability of 0.1 °C.

### Data Processing

The data of 11 out of the 14 participants were completely collected and analysed. The other three participants failed to complete effective data collection due to premature discharge of the capsule or loss of data. First, the raw CBT data were processed for outlier elimination based on special event markers. After that, the CBT data were analysed by the cosine method as Eq. (4) [36]. The data processing of curve fitting was conducted by the curve fitting toolbox in MATLAB.

4 $$y = A \times \cos (2\pi \times (x - \phi )/T),$$

where $A$ represents the amplitude, $\phi$ represents the acrophase, and $T$ represents the period of the CBT data. Participants could obtain a series of fitting curves of CBT under each scheme. According to the curve, the time at the minimum point of CBT was obtained, which was regarded as CBT min for the next analysis step.

On this basis, the CBT min of scheme C was set as the baseline data of the participant. *t*-test was performed between scheme C and the other five schemes separately to test whether the difference between them is significant. If these differences are significant, the CPS of the other five schemes was obtained by subtracting the baseline CBT min result from the experimental data. The CPS data of H-M, H-A, H-E and H-MA were processed by analysis of variance (ANOVA) to investigate the effects of lighting exposure timing and duration, and the data of H-MA and L-MA were tested by *t*-test to demonstrate the spectral effects. Finally, multiple linear regression (MLR) was employed to construct the CPS phenotype model. All the analyses above were processed by IBM SPSS Statistics 22.

## Results

Firstly, the CBT-min data under all schemes satisfy the hypothesis of homogeneity of variance, and the *t*-test results showed significant differences between scheme C and the other five schemes (*p* < 0.05). Hence, the CPS of these five schemes were obtained by subtracting the baseline CBT-min result from the scheme C.

### Time Factor: Timing and Duration

**Fig. 3:**
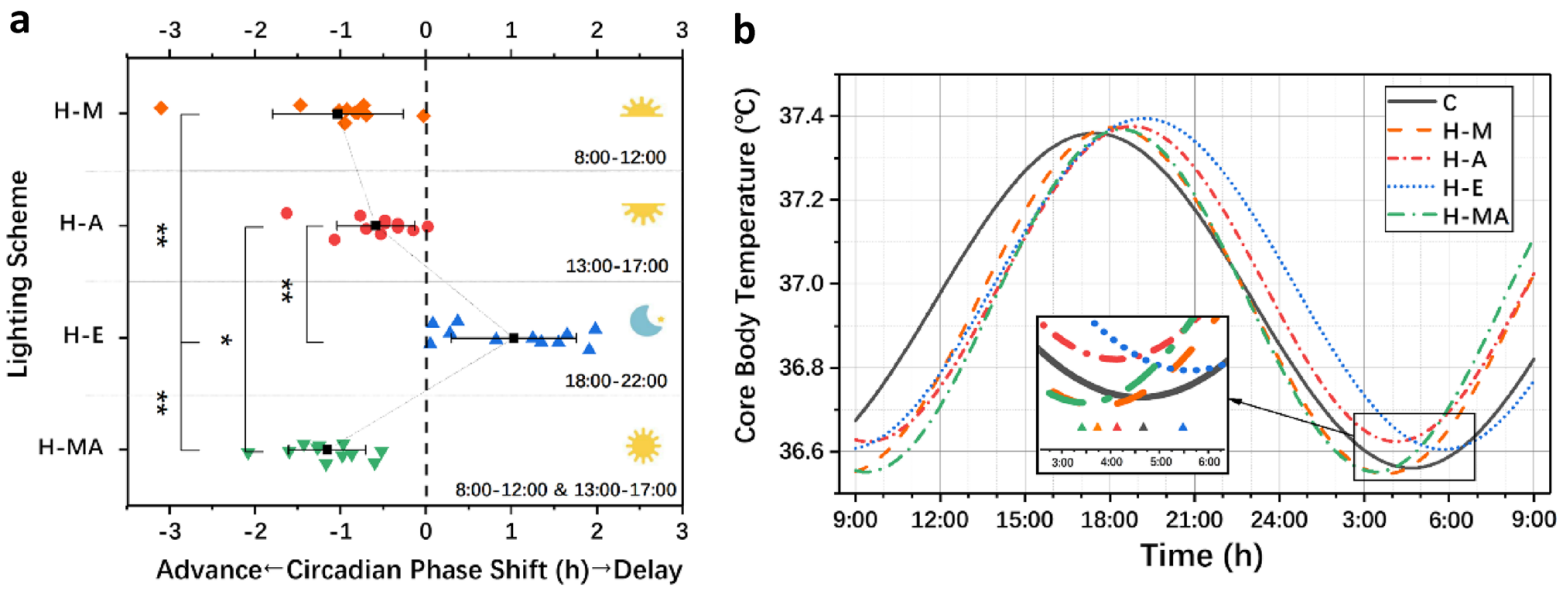
The results of CPS and CBT data under different lighting schemes:** a** Mean and standard deviation of CPS and ANOVA result and** b** Fitted CBT data and CBT min *p < 0.05, **p < 0.01

ANOVA was performed for the CPS data of H-M, H-A, H-E and H-MA to test the effects of light exposure timing and duration on circadian rhythm. Levene’s test results showed that the data structure satisfies the hypothesis of homogeneity of variance; thus, ANOVA can be conducted. The ANOVA results of the CPS showed a significant effect of different timing and duration schemes on the CPS (*F*_3,40_ = 29.079, *p* < 0.001). The specific CPS data in each scheme and the ANOVA results are listed in Table 2 and Fig. 3a. Figure 3b is an example of the fitted CBT data for each scheme.

**Table 2:** The mean and standard deviation (SD) of CPS data in the H-M, H-A, H-E and H-MA schemes and ANOVA results

Measurement	Relative value of CBT min (Intervention-baseline)								ANOVA	
	H-M (*N* = 11)		H-A (*N* = 11)		H-E (*N* = 11)		H-MA (*N* = 11)			
	Mean	SD	Mean	SD	Mean	SD	Mean	SD	*F*	Sig
CPS (h)	−1.032	0.764	−0.588	0.456	1.027	0.734	−1.158	0.450	29.079	** < 0.001**

The least significant difference (LSD) post hoc test was employed to inspect the difference between every two schemes. The results showed that the CPS of H-E was significantly larger than that of the other three schemes (*p* < 0.001), indicating that the H-E scheme could significantly delay the circadian phase compared with the other schemes. It also demonstrated that the timing of light exposure is an essential factor of the phase shift direction. That is, compared to dim light, light exposure during the daytime, which is synchronized with daylight, could advance the circadian phase of the human body, thus counteracting the endogenous delay of the human circadian phase. However, light exposure at night, which departs from the daylight schedule, could delay the circadian phase of the human body, thereby disturbing the stability of the circadian rhythm. In addition, compared with the H-A scheme, H-MA showed a significant difference (*d* = 0.570, *p* = 0.037) in advancing the circadian phase, which indicated that the duration has a significant influence on the magnitude of the phase shift. Light exposure for a longer time could accumulate more photobiological effects on the human body, thus triggering a greater impact on the CPS. Furthermore, the post hoc analysis (α = 0.05; two-tail) of the ANOVA was calculated by G*Power (Faul et al. 2007, 2009) with 11 subjects showed that the power of the statistical test was equal to 0.98, which revealed that although there is individual difference, the average results of the subjects has a stable statistical meaning.

### Effective Circadian Stimulus Level Factor

**Fig. 4:**
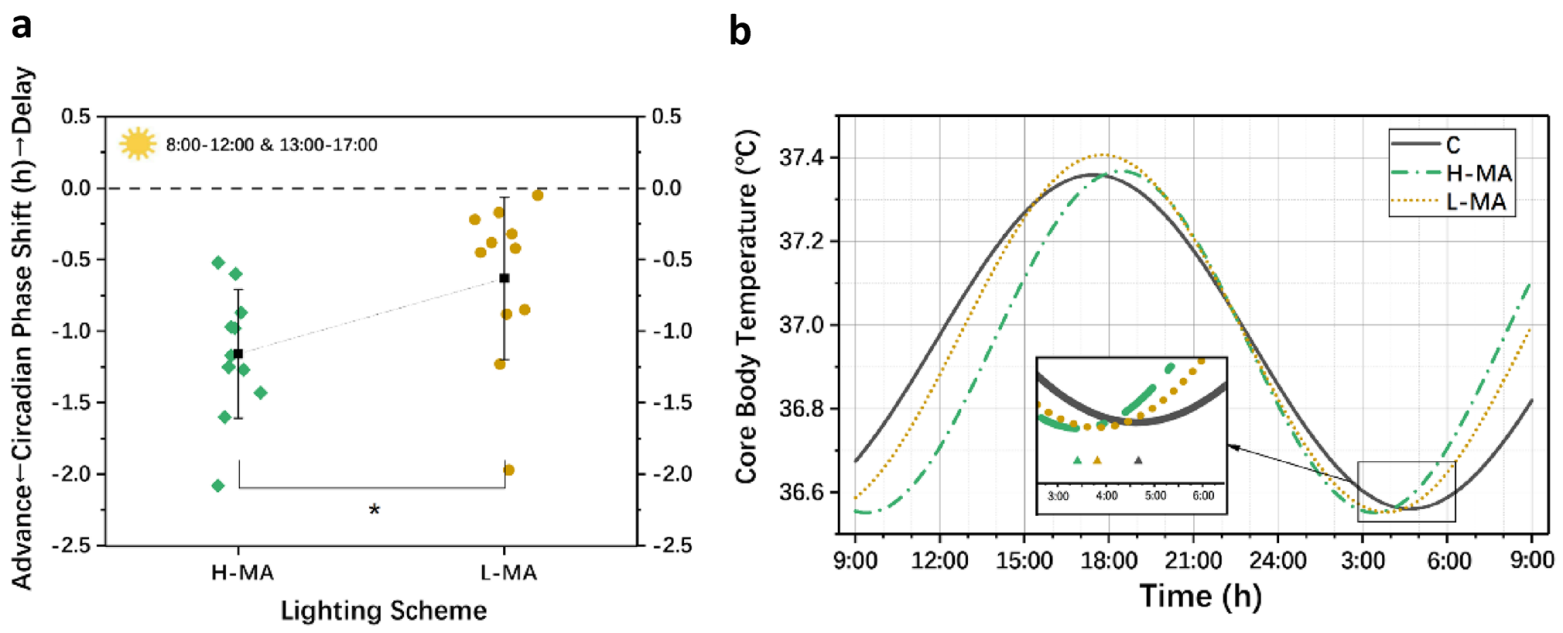
The results of CPS and CBT data under two lighting schemes:** a** Mean and standard deviation of CPS and *t*-test result and** b** Fitted CBT data and CBT min *p < 0.05

A *t*-test was used to analyse the CPS data between H-MA and L-MA to investigate the effects of the circadian stimulus level factor on CPS. According to the result of Levene’s test, the variance of the CPS showed homogeneity; thus, the *t*-test could be conducted. The results revealed that different circadian stimulus schemes showed a significant difference in the CPS (*T*_20_ = −2.415, *p* = 0.025), which indicated that the level of the circadian stimulus is also an important factor for the magnitude of the CPS. This means that without changing the illuminance and colour temperature of the light, various circadian stimulus levels caused by spectral modulation could also trigger different adjustment effects on the CPS. In other words, compared with the traditional illuminance and colour temperature, the circadian stimulus level or melanopic equivalent illuminance based on the non-visual pathway are effective indicators to describe the light stimulus level for the human circadian phase. The specific CPS results in these two schemes and the result of the *t*-test are listed in Table 3 and Fig. 4a. Figure 4b is an example of fitted CBT data for each scheme. The post hoc test of *t*-test effect size showed that the statistical power equalled 0.76 with acceptable reliability.

**Table 3:** The mean and standard deviation (SD) of CPS data in the H-MA and L-MA schemes and *t*-test results

Measurement	Relative value of CBT min (Intervention-baseline)				*t*-test	
	H-MA (*N* = 11)		L-MA (*N* = 11)			
	Mean	SD	Mean	SD	*T*	Sig
CPS (h)	−1.158	0.449	−0.630	0.568	−2.415	**0.025**

## Modelling of the Diurnal Circadian Lighting Accumulation (DCLA) and Circadian Phase Shift (CPS)

As assumed in the introduction, time factors, including time point and duration, as well as the effective circadian stimulus level, have been shown to have significant impacts on the shift amplitude and direction of the circadian phase, as described in “Results” section. Next, the DCLA model was constructed by the time factor and effective circadian stimulus level factor. In this section, the time factor and effective circadian stimulus level factor would be integrated to define the DCLA. On this basis, the DCLA-CPS prediction model was proposed as a sigmoidal curve. Then, the experimental data of this study and the previous researches were introduced to verify the DCLA-CPS model.

### Important Definition for DCLA Model

#### Diurnal Phase Shift Curve

**Fig. 5:**
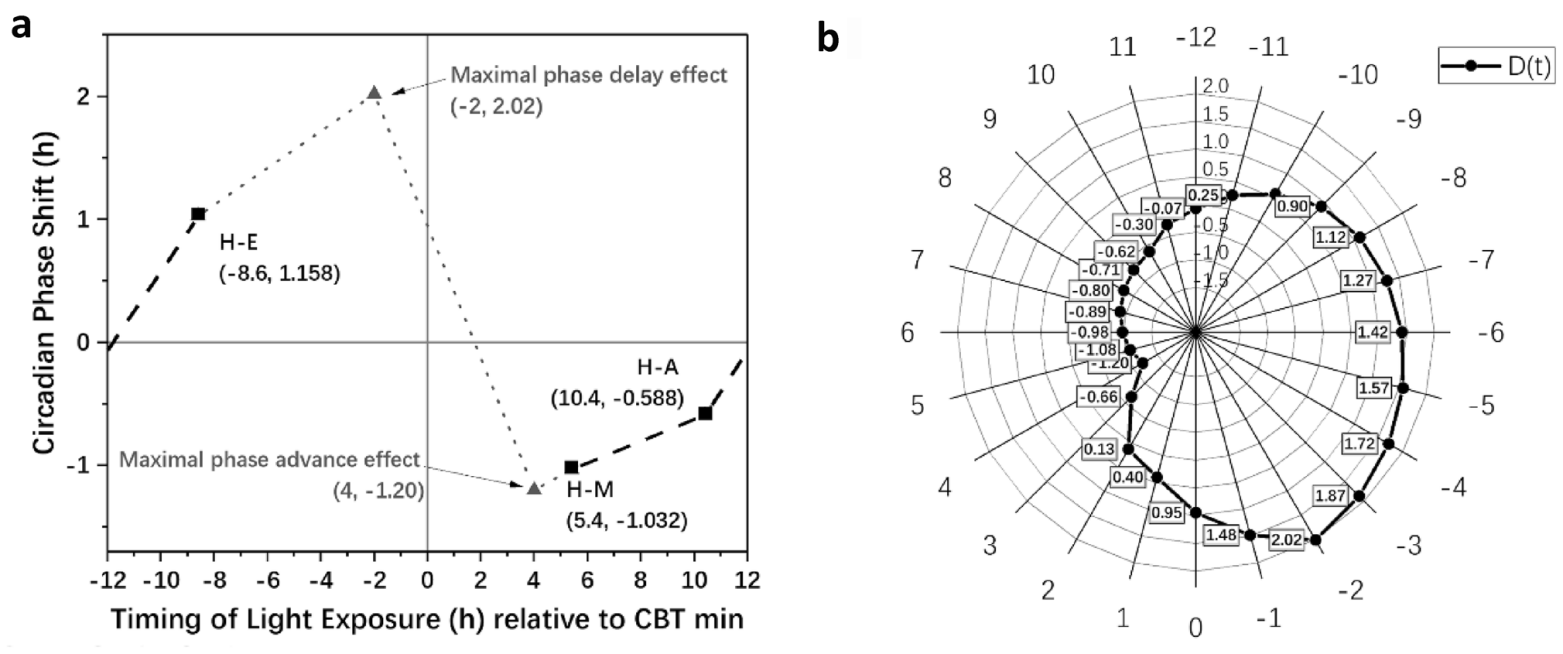
The diurnal phase shift curve.** a** The raw data of *D*(*t*) and **b** *D*(*t*) at different times. The squares represent the data obtained from this study, and the black dashed line in the curve was plotted by the data from this study; the triangles represent the data referenced by a previous study (Minors et al. 1991; St Hilaire et al. 2012), and the grey dotted line in the curve was plotted by the referenced data. The horizontal axis of **a** and the circumferential direction of **b** represent the timing of light exposure relative to CBT min, “−” means the time before CBT min, and “+” means the time after CBT min. The vertical axis of **a** and the axis of **b** represent the relative diurnal circadian phase shift, “−” means phase advance, and “ +” means phase delay

According to the experimental results, the light exposure at different timing showed different effects on the magnitude and direction of the CPS. Therefore, it was necessary to construct a diurnal phase shift curve (*D*(*t*)), which was used to describe the response vector of the light exposure at different timing on the CPS. The timing of light exposure in *D*(*t*) was defined as the midpoint of the light exposure relative to CBT min in a range from −12 to 12 h, which is a classical metric in a previous study (Minors et al. 1991). To get a preliminary quantification of *D*(*t*), the results of this research and previous researches were introduced to make assumptions about the *D*(*t*) curve. According to the mean CBT min (4:36) in the C scheme, the light exposure timing of the H-M (midpoint-10:00), H-A (midpoint-15:00) and H-E (midpoint: 20:00) schemes were equal to 5.4, 10.4 and −8.6 h, respectively. Hence, we could provide three points on the diurnal phase shift curve based on the phase shift results of H-M, H-A and H-E. As only the data from 6 to −8 h were covered by our experiment in this curve, we explored a previous study to complete the curve. The data from Minors et al. (1991) showed that the maximal phase advance effect appeared approximately 4 h after the CBT min, and the maximal phase delay effect appeared 2 h before the CBT min. In addition, t Hilaire validated that a 1-h bright white light (4000–8000 lx) exposure could reset the circadian rhythm with a maximal phase delay shift of 2.02 h and phase advance shifts of 1.20 h (St Hilaire et al. 2012). Considering the regulation of the CPS verified by previous research (Hou et al. 2021a), long-term exposure to mild light has approximately equivalent effects to short-term bright light. Therefore, the effect of the 4-h mild light exposure (1000 lx, CS = 0.55) at different time points in this study on CPS can be regarded as the maximal phase shift caused by the 1-h bright light at that time point. On this basis, the points of (4, −1.20) and (−2, 2.02), which were obtained by referring to the magnitude of the phase shift in the study of St Hilaire et al. (2012) and the time of maximal effect in the study of Minors et al. (1991), were used as the supplemented points of the time period not covered in this study. A total of five points and the diurnal phase shift curve (*D*(*t*)) are shown in Fig. 5a, b.

The value corresponding to the time in *D*(*t*) only represented the magnitude and direction of the light exposure with different timing schemes on the CPS as a quantified result of the time factor. In addition, *D*(*t*) was a curve composed of discrete points, with the step length of 1 h. The specific value of *D*(*t*) are shown in Fig. 5b.

#### Effective Circadian Stimulus Level

As shown in “Results” section, the effective circadian stimulus level showed significant effects on the CPS, even when the CCT and illuminance remained constant. Hence, the lighting stimulation level metrics developed based on the non-visual response pathway should be considered. A previous study demonstrated that CL_A_ (Rea and Figueiro 2018) and *E*_z_ (Lucas et al. 2014) are both good predictors for the CPS, considering the photobiological effect (Hou et al. 2021a). The abovementioned three metrics (CS, CL_A_ and *E*_z_) were good tools to describe the effective circadian stimulation level (ECS). For their specific calculation methods, see the Eq. (1–3) in the introduction section. The ECS that people with light exposure in a biological day − 12 to 12 h was characterized as ECS(*t*). To facilitate sampling and calculation, the sampling interval of this curve was also 1 h, which is synchronized with *D*(*t*). ECS(*t*) was used to contribute to the calculation of the DCLA. CS, CL_A_ and *E*_z_ were used as effective methods to describe ECS(*t*). According the fitting performance of the DCLA-CPS model, one would be recommended among the CS, CL_A_ and *E*_z_ as the parameter to describe ECS(*t*).

#### The Definition of DCLA

As stated above, the effects of the time factor on the CPS were quantified as *D*(*t*) to describe the effects of the light exposure on the CPS at a specific timing. In addition, ECS(*t*) was defined to characterize the stimulus level of light at different timings. On this basis, DCLA was established by accumulating the diurnal circadian stimulus factor (ECS(*t*)) and phase shift curve (*D*(*t*)), as shown in Eq. (5).

5 $${\text{DCLA}} = \sum\limits_{t = - T}^{T} {{\text{ECS}}(t)} \cdot D(t),$$

where $- T$ represents − 12, which means the timing of 12 h before the CBT min; $T$ represents 12, which means the timing of 12 h after CBT min; ${\text{ECS}}(t)$ is the diurnal light stimulation curve; and $D(t)$ is the diurnal phase shift curve. DCLA is the sum of the product of CSD(*t*) and *D*(*t*) in each hour of the 24 h biological day, which is the diurnal circadian lighting accumulation.

### The Establishment of the DCLA-CPS Model

To predict the CPS through the sampling of DCLA, it was necessary to further construct the DCLA-CPS model. Considering the response compression of the effects of light exposure on circadian rhythm, CPS would not continue to rise with an increase in the accumulation of light, and there were limitations in both the phase advance and delay shifts (Chang et al. 2012). Hence, the Boltzmann function was employed to fit the impacts of DCLA on CPS, which demonstrated that this logistic was a proper method to fit illuminance‐response CPS data in a previous study (Zeitzer et al. 2000a), and the specific function is shown in Eq. (6)

6 $${\text{CPS}} = A2 + \frac{A1 - A2}{{1 + {\text{e}}^{{\frac{{{\text{DCLA}} - x_{0} }}{d}}} }},$$

where CPS represents the circadian phase shift, A1 represents the lower limit of the CPS (maximum advance shift), A2 represents the upper limit of the CPS (maximum delay shift), and x_0_ and d work together on the slope and direction of the S-shaped curve.

### The Validation and Parameter Determination of the DCLA-CPS Model

To validate the DCLA-CPS model, five experimental conditions in this study and the results of previous studies were introduced to do an integrated analysis. The inclusion criteria of the previous studies were used to explore the effects of light intervention on human CPS, and the exclusion criteria were as follows: (1) no clear light exposure description: time periods, durations and illuminance levels and (2) no detailed circadian phase information (melatonin or CBT phase). The specific information of the included studies is listed in Table 4. Because the spectra in the other three studies were not reported, based on the description of CCT in the research, CIE D65 was employed to calculate the lighting parameters in the study of Carrier and Dumont (1995) and Dewan et al. (2011), and CIE F32 was used to calculate the specific parameters in Chang's study (Chang et al. 2012). The ECS value was calculated by *E*_z_, CL_A_ and CS, and the DCLA could thus be contributed by these three indexes. When the light intervention covered multiple hours, the minimum time interval of ECS(*t*) was set to 1 h, which is consistent with the $\Delta t$ of *D*(*t*) in Fig. 5b. In this way, a long duration light exposure is broken down in the time dimension, and the corresponding *D*(*t*) is assigned every hour in the light exposure duration. Then, an accumulation operation was performed to calculate the DCLA.

**Table 4:** The DCLA and CPS data in related studies

Source	Scheme abbreviation	Time factor-light exposure	Effective circadian stimulus (ECS) for intervention			Diurnal circadian lighting accumulation (DCLA)			Circadian phase shift (CPS)
			*E*_z_ (lx)	CL_A_	CS	*E*_z_ (lx)	CL_A_	CS	
This study	H-M	3.4–7.4	937.84	1177.83	0.55	−3754.32	−4715.03	−2.20	−1.03
	H-A	8.4 to −11.6	937.84	1177.83	0.55	−2009.22	−2523.38	−1.18	−0.59
	H-E	−10.6 to −6.6	937.84	1177.83	0.55	3046.45	3826.03	1.79	1.03
	H-AM	3.4–7.4 and 8.4 to −11.6	937.84	1177.83	0.55	−5763.54	−7238.41	−3.38	−1.16
	L-AM	3.4– 7.4 and 8.4 to −11.6	520.09	353.97	0.35	−3196.24	−2175.34	−2.15	−0.63
Carrier and Dumont (1995)	Morning	2.9–7.9	10,488	20,521	0.69	−49,286.46	−96,434.73	−3.24	−1.23
	Evening	−10.7 to −5.7	10,488	20,521	0.69	46,786.40	91,543.07	3.08	1.62
	Afternoon	7.2 to −11.8	10,488	20,521	0.69	−33,124.52	−64,812.01	−2.18	−0.48
Chang et al. (2012)	0.2 h	−3.2 to −3	5594	9278	0.68	1921.82	3187.46	0.23	0.67
	1.0 h	−3.5 to −2.5	5594	9278	0.68	10,027.27	16,630.86	1.22	1.15
	2.5 h	−4.2 to −1.7	5594	9278	0.68	25,199.05	41,794.20	3.06	1.89
	4.0 h	−5.0 to −1.0	5594	9278	0.68	40,127.10	66,553.32	4.88	2.25
	6.5 h	−6.5 to 0.0	5594	9278	0.68	59,909.50	99,363.67	7.28	2.65
Dewan et al. (2011)	2000 lx	−4.5 to −1.5	1243	2061	0.61	6686.25	11,086.38	3.28	0.77
	4000 lx	−4.5 to −1.5	2486	4124	0.66	13,372.50	22,183.51	3.55	1.46
	8000 lx	−4.5 to −1.5	4972	8274	0.68	26,745.01	44,506.88	3.66	2.04

As the response compression of light exposure on circadian rhythm (Chang et al. 2012), the Boltzmann function was employed to fit the impacts of DCLA on CPS. Fitting was performed between CPS and DCLA, which were calculated using different parameters, including *E*_z_, CL_A_ and CS. The fitting results are listed in Table 5.

**Table 5:** The Boltzmann fitting results of DCLA and CPS

Parameter	Formula	*A*1	*A*2	*x*_0_	*d*	Adjusted *R*^2^	Residual sum of squares
DCLA (*E*_z_)	$y = A2 + \frac{A1 - A2}{{1 + {\text{e}}^{{\frac{{x - x_{0} }}{d}}} }}$	−1.10	2.01	3268.28	4194.69	0.8914	0.1891
DCLA (CL_A_)		−1.10	2.00	5619.43	6551.46	0.8690	0.2280
DCLA (CS)		−1.58	2.65	0.72	2.11	0.9320	0.1184

**Fig. 6:**
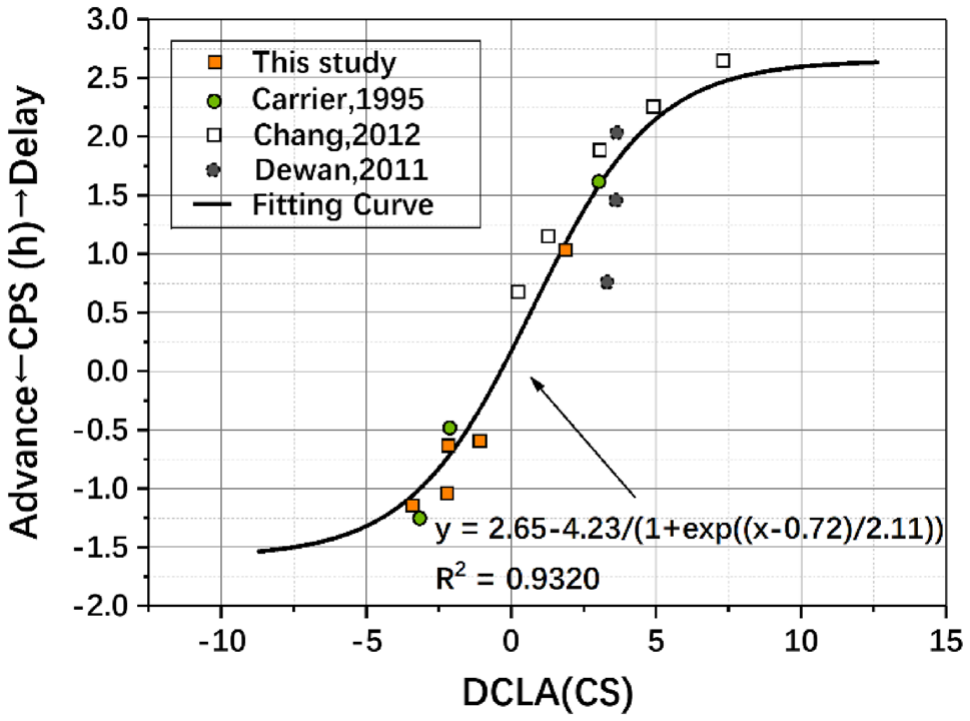
The Boltzmann fitting curve of DCLA to CPS, based on data in Table 4 (Carrier and Dumont 1995; Dewan et al. 2011; Chang et al. 2012)

The results in Table 5 show that the DCLA calculated by CS has the highest fitting degree (*R*^2^ = 0.9320) and the smallest residual sum of squares (RSS = 0.1184) to CPS among the three indicators. Therefore, CS was finally selected as the light level parameter for calculating ECS(*t*); that is, ECS(t) was the curve of light stimulation recorded by the CS value within 24 h (from −T to T) of diurnal stimulation. The fitting functions of the CPS and DCLA are shown in Eq. (7) and Fig. 6, which proved that the hypothesize of a sigmoidal model between DCLA and CPS well describe their relationship.

7 $${\text{CPS}} = 2.65 - \frac{4.23}{{1 + {\text{e}}^{{\frac{{{\text{DCLA}} - 0.72}}{2.11}}} }},$$

where CPS represents the circadian phase shift, its magnitude represents the magnitude of the phase shift, positive or negative represents the direction of the phase shift, a positive CPS represents the circadian phase delay, and a negative CPS represents the circadian phase advance. DCLA is calculated by Eq. (5).

## Discussion

In this study, the timing, duration and ECS of light exposure were set as the variables and investigated through a within-subject experiment. Based on the experimental results in two dimensions, a DCLA-CPS model was proposed in the form of Boltzmann function, and validated by the experimental data. Further analysis of each dimension and discussion based on previous research were as follows.

The analysis results of the time factor showed a significant effect of different timing and duration schemes on the CPS (*F*_3,40_ = 29.079, *p* < 0.001). The CPS value of the H-E scheme was significantly delayed compared with that of the other three phase advance schemes (*p* < 0.001), which indicated that the timing of light exposure is a critical factor for the phase shift direction. Light during the daytime (H-M, H-A) could advance the circadian phase, while light at night could delay the phase. These results were consistent with the conclusion of Carrier and Dumont's study (Carrier and Dumont 1995) that the bright light exposure in the "morning group" and "afternoon group" showed an advance of 1.23 and 0.5 h, respectively, and that of "evening group" showed a delay of 1.62 h. In summary, different timing schemes of light exposure triggers different CPS action vectors with individual direction and amplitude. In addition, although with a double light exposure duration showed the best performance in phase advance, the H-MA scheme did not obtain twice of the CPS results compared with the H-M and H-A schemes, which shows that the influence of duration on CPS is not linear (Chang et al. 2012) but depends on each light exposure timing. Moreover, due to the influence of individual differences, for some people with an earlier circadian time of 0, the H-A scheme may include the effects of phase advance (before *T* = 12 (−12) h) and delay (after *T* = 12 (−12) h) at the same time. Therefore, this situation caused an offsetting influence of the phase advance effect in the H-MA scheme. This may be the reason why there is no significant difference between H-M and H-MA. Therefore, a diurnal phase shift curve (*D*(*t*)) regarding light exposure timing and individual circadian phase should be used as a more accurate dose metric, rather than that just based on the duration of exposure. In addition, compared with the PRC proposed in Minors’ study in 1991, the *D*(*t*) in this study had a similar trend to the PRC and remained consistent in the magnitude and timing of the phase shift extreme. However, in other periods (morning, afternoon and evening), *D*(*t*) was more dependent on the data at the three time points tested in this research. Linear interpolation was used to predict the CPS instead of taking the average phase shift of multiple studies, which makes the response curve smoother and avoids the strange inflection point caused by the difference from various studies. However, more data are still needed to verify the accuracy of *D*(*t*).

The analysis results of the ECS also showed a significantly different effect on CPS (*T*_20_ = −2.415, *p* = 0.025). This is a fascinating result because people usually adjusted the circadian phase by changing the light intensity (Dewan et al. 2011; Hou et al. 2021a) or CCT (Crowley and Eastman 2017) in past studies. This research proved that without changing the light intensity and CCT, a similar effect could be achieved through sophisticated spectral modulation. Although some scholars have proven that the secretion of melatonin (Souman et al. 2018) and sleep quality (Hou et al. 2021b) could be affected by spectral modulation in the past, this is further proof that the effect of spectral modulation was also effective for CPSs. Hence, light parameters that could describe the photobiological effects produced by the non-visual pathway should be considered to describe the influence of light exposure on the circadian rhythm, and the diurnal effective circadian light stimulation level (ECS(*t*)) is an important concern.

The DCLA model established based on the above two important dimensions that affect the CPS showed a good fitting degree with the CPS (*R*^2^ = 0.9320, RSS = 0.1184). This model not only confirmed the law shown in this study but also showed a good fitting degree for several early representative studies (Carrier and Dumont 1995; Dewan et al. 2011; Chang et al. 2012). Compared with the earlier PRC (Minors et al. 1991), the *D*(*t*) curve in this study was based on a broken line drawn by five characteristic points and gives the timing effect of the relative rhythmic phase shift, which provided a clearer quantitative description of the time effect of light, as shown in Fig. 5b. In addition, the parameters used to describe the light ECS in the model were selected carefully. The Boltzmann fitting results proved that CS has the best fitting degree compared to *E*_z_ and CL_A_. This result is different from the indicators used in the previous DLMO shift model (Hou et al. 2021a). The better fitting effect of CS may be caused by the construction process of CS, which has considered the response compression effect of the light level (Rea and Figueiro 2018).

Furthermore, the prediction results of the DCLA-CPS model approached the conclusions of previous studies. That is, the upper limit of the phase delay is 2.65 h (Chang et al. 2012), the upper limit of the phase advance is 1.58 h (Carrier and Dumont 1995; Hou et al. 2021a), and if there is no external light intervention or the effective result of light intervention, then the circadian rhythm phase would be the natural delay of 0.18 h per day (Czeisler et al. 1999).

In summary, the DCLA-CPS model is an integrated model that not only describes the combined effects of the two factors of light exposure timing and duration through the time response function (*D*(*t*)) but also introduces the non-image function indicators of light to accurately quantify the effect of light intensity on the CPS. In addition, the DCLA-CPS model shows a good fitting degree (*R*^2^ = 0.9320, RSS = 0.1184) when validated by the outcomes in this research and the data from previous studies. Thus, this model is worthy to be recommended to predict the CPS caused by diurnal light exposure.

However, there was also some limitation in this study. The preliminary establishment of the DCLA-CPS model was based on an integrated analysis of the results of this study and the other three studies. Although it currently showed a good predictive effect, the universality and accuracy of *D*(*t*) and DCLA-CPS model requires more researches to verify and optimize in the future. In addition, the participants in this study were mostly young people; thus, the age factor was not considered in the model. In addition, different populations showed different gene expression in terms of light-sensitive characteristics (Kolker et al. 2003; Daneault et al. 2016). In the future, more individual physiological characteristics will be included in the study, such as age groups and light-sensitive genotypes.

## Conclusions

In this study, a strict light exposure experiment with different timing schemes, durations and effective circadian stimulus levels was performed to explore the effects of different dimensions of light exposure on the physiological CPS of the human body. The results showed that the effect of different timing and duration schemes and different circadian stimulus levels showed a significantly different effect on the CPS. On this basis, a DCLA model was established that could well predict the CPS (*R*^2^ = 0.9320, RSS = 0.1184). This work provides strong support for rationally arranging the light level at different times of the day.

## Data Availability

All data generated or analysed during this study are included in this published article.

## Abbreviations

CBT: Core body temperature
CC: Control condition
CCT: Correlated colour temperature
CL_A_: Circadian light
CPS: Circadian phase shift
CRI: Colour rendering index
CS: Circadian stimulus
DC: Dim condition
DCLA: Diurnal circadian lighting accumulation
DLMO: Dim light melatonin onset
ECS: Effective circadian stimulus level
Ev: Vertical illuminance
*E*_z_: Melanopic illuminance
HIC: High intervention condition
H-M: High intervention in the morning
H-A: High intervention in the afternoon
H-E: High intervention in the evening
H-MA: High intervention in the morning and afternoon
ipRGCs: Intrinsically photosensitive retinal ganglion cells
LIC: Low intervention condition
L-MA: Low intervention in the morning and afternoon
SPD: Spectral power distribution
